# Nature and Art

**Published:** 1861-03

**Authors:** N. S. Davis


THE CHICAGO
MEDICAL EXAMINER

VOL. II. MARCH, 1861. No. 3

### ORIGINAL CONTRIBUTIONS.

*Nature and Art. Their relative influence in the management of Diseases. Are they antagonistic or co-operative? An Essay read to the Chicago Medical Society, Oct. 19th, 1860.* By N. S. DAVIS, M. D.

During the last ten or fifteen years much has been said and written concerning the curative powers of Nature; as though she was an actual *entity*—a fair Goddess of health, ruling over the animal economy with the ubiquitous power to meet disease at every point.

And while nature has been thus personified, and exalted as an active curative power, *Art* has been caricatured as little else than an indiscriminate exhibition of harsh and deleterious drugs. Drs. Forbes and Bigelow, took the lead in this modern effort to deify nature, and it has been followed up, both in and out of the profession, until we can hardly take up a popular address, an introductory lecture, or an essay in the pages of a Medical Journal, without finding the curative powers of *nature* extolled, and the assumed *over-drugging* of the human race under the direction of art, soundly berated.

One of the latest and most noted productions of this kind, is the annual address, delivered before the Massachusetts Medical Society, by Dr. Oliver Wendell Holmes, of Boston; entitled, "Currents and Counter Currents in Medical Science." In perusing this kind of literature, two questions always arise in the mind, namely: 1st, what is meant by *Nature* as used in medical language; and 2d, what are the true relations between nature and art? Most of that class of writers who extoll nature and depreciate art, use the two words as though they were necessarily antagonistic, but make no attempt to define either of them. Dr. Holmes, however, has so far deviated from the beaten path, as to define both terms as follows: "*Nature*, in medical language, as opposed to Art, means trust in the reactions of the living system against ordinary normal impressions."

"*Art*, in the same language, as opposed to Nature, means an intentional resort to extraordinary abnormal impressions for the relief of disease."

"*Disease*, dis-ease---disturbed quiet, uncomfortableness, means imperfect or abnormal reaction of the living system and its more or less permanent results."

"*Food*, in its largest sense, is whatever helps to build up the normal structures, or to maintain their natural actions."

"*Medicine*, in distinction from food, is every unnatural or noxious agent applied for the relief of disease."

These definitions emanating from a high literary source, and directly from what is claimed as the head quarters of conservatism in medicine, may reasonably claim a little examination. It will be seen by the first paragraph quoted that *Nature*, in medical language "means *trust*," that is, reliance, faith; therefore, according to Dr. Holmes, *nature* is merely a *mental* act, capable of being exercised either by the patient or the physician, or by both. "*Trust* in the reactions of the living system against ordinary normal impressions," constitutes *nature*. Surely, the definition is sufficiently simple, but can anything be more manifestly absurd? It is probable, however, that Dr. Holmes meant to convey the idea, that *nature* is the reaction of the living system against ordinary normal impressions, instead of *faith* or *trust* in such reaction. But while his language makes nature consist in a mere mental act of faith, *Art* consists in the production of "extraordinary abnormal impressions;" and disease simply "means imperfect or abnormal reaction of the living system," &c. Surely, the author of definitions so lucid, and so comprehensive, should have a monument erected to his memory!

Nature is the normal *reaction* of the living system.

Disease is imperfect or abnormal *reaction* of the living system.

*Art* is the production of abnormal *impressions* on the living system. But what is meant by the words, "*reaction*" and the "*living system*"? According to lexicographers, *re-action*, means action restored, renewed, re-established; and necessarily pre-supposes a previous diminution or suspension of action.

But can "ordinary normal impressions" produce anything more than simply, *action*? If not, how can *nature* be synonymous with *re-actions*? It was no part of our intention, however, to waste time in criticising the absurd definitions of Dr. Holmes. On the contrary we quoted them for the purpose of exhibiting more clearly, certain fundamental errors that are common to the whole class of writers, represented by Holmes, Bigelow, Forbes, &c. The first and most important of these errors, is the assumption that *nature* and *art*, as used in reference to the treatment of disease, are antagonistic or opposed to each other.

The second, is the assumption, that all medicines are "unnatural or noxious agents," producing "extraordinary abnormal impressions" on the living system.

The third, is the assumption that whatever is injurious or debilitating to the human system in health, is equally so to the same system affected by disease.

These three assumptions, constitute the foundation on which is based all the reasonings and deductions of "Young Physic"—"Nature and Art in the cure of Disease"—and a host of minor productions in the form of Addresses, Essays, &c. To expose the fallacy of these assumptions and point out, what we deem to be, the true relations of *nature* and *art* in the cure of disease, are the special objects of this paper.

*Nature* is a word which occurs so frequently in the medical writings of all ages, that its import, or the ideas sought to be conveyed by it, ought to be clearly understood. It is generally used in such a manner as to indicate, not only, an active, but a more or less intelligent power.

Hence, the common expressions, "Powers of Nature"—"efforts of nature"—"curative powers of nature"—"vis medicatrix naturæ"—&c., &c. If we take the plain import of the language used, we should necessarily receive the idea that the living physical organization of man, is possessed of a definite property or power, not only capable of resisting morbid impressions, but capable also, of making active and well directed *efforts* to remove morbid action when already established.

The *Archæus* of Van Helmont, did not more clearly represent the idea of a distinct presiding power in the animal economy, than does the word *nature*, as used in the medical literature of the present day. But is there any such definite property or power? We doubt whether any enlightened physician of the present day, would answer in the affirmative. What, then, is the proper meaning of the word Nature as used in medicine.

A rational answer to this question, must necessarily precede any attempt to point out the true relation between nature and art. If the word has any definite meaning in medical discussions, it must refer to the elementary or vital properties of the organized structures, and the primary functions resulting from the action of natural stimuli on those properties. Viewing man as a living physical being, without reference to mind, we should say that every cell and fibre of his organized structures, are endowed with two elementary properties. The first we call *susceptibility*, or that property which renders the primary organized structures capable of being acted upon in a given direction. The second, we call *vital affinity*, or that property by which the particles of organized matter are made to arrange themselves in peculiar and definite forms, constituting the various primary structures of the body. By one class of writers the first of these properties has been confounded with nerve sensibility; and by another, both have been confounded under the vague expressions—life force—vital force—vitality—principia vitæ, &c. But a careful logical analysis of the phenomena of organization and life, will show that these two distinct properties not only exist, but that they are also primary and inherent in all living matter, without any reference to nerve structure or function. In the absence of active stimuli, they are capable of remaining dormant for an indefinite time, as seen in the seeds and germs of plants and the ovum of animals. But no sooner is the appropriate stimulus of caloric, moisture, and nutritive material presented, than a *susceptibility* is manifested by active changes; and a *vital affinity*, by the peculiar and definite arrangement of particles resulting from such changes.

Each of the primary structures of the human body, such as the nervous, muscular, fibrous, vascular, and secretory being endowed with these properties, are capable of manifesting certain phenomena or results which we call elementary functions.

Thus the nerve structure is capable of receiving and transmitting impressions; the muscular of contractions; the fibrous of elasticity; the vascular of circulating fluids; and the secretory of selecting materials and forming new compounds, called secretions. Thus by an analytical study of organized living matter, we find two elementary properties common to all such matter; and as a result of these we find each definite structure endowed with a capacity for certain actions called elementary functions. They may be tabulated thus:

| | | |
|---|---|---|
| Elementary Properties. | Susceptibility, | Common to all living matter. |
| | Vital Affinity, | |

| | | | |
|---|---|---|---|
| Elementary functions. | Sensibility and Transmissibility, | | Belonging to nerve structures. |
| | Contractility— | belonging | to muscular structures. |
| | Elasticity— | do | fibrous structures. |
| | Circulation— | do | vascular structures. |
| | Secretion— | do | secretory structures. |

From these elementary properties and functions, aided by the natural stimuli and appropriate material to act upon, result all the complex phenomena of physical life and organization.

Now, the sum total of these properties, structures and functions, as they are presented in the living animal body constitutes *nature*. And when we speak of the *powers* or *efforts* of nature, we can mean nothing more or less than the actions resulting from the performance of one or more of these functions.

Some of these actions have for their object the reception, assimilation, distribution, and appropriation of material for growth and reparation. Others, for the disintegration and removal of waste or hurtful material from the system. The former are styled nutritive actions, and the latter excretory or depurative. It is chiefly through the latter class of actions or functions that those changes are effected, which in common parlance are styled efforts of *nature* to prevent or cure disease. Thus if food is taken in too large quantity or of bad quality, it often irritates the mucous membrane to such an extent as to produce either vomiting or purging, by which the offending material is expelled, and *nature* is said to have relieved herself. Again, if matter foreign and hurtful in its nature, passes the digestive organs and enters the blood, it is often speedily separated and passed off by the skin, kidneys, and lungs, thus preventing the establishment of morbid action or rendering it evanescent. This is another mode of displaying what is called the curative efforts of nature.

But some of the excretory organs are so constituted that the increased action of one may temporarily compensate for the diminished action of another. Thus, a diminished action of the skin from undue exposure to cold, is often accompanied by sufficient increased action of the kidneys to prevent hurtful accumulations of effete matter in the blood. These are also denominated *efforts* of *nature.* There are also various other processes by which morbid actions are suppressed or diseases limited in their duration, that are ranked as efforts of *nature*, but the rationale of which is less generally understood.

Thus when an irritant acts upon the nervous system so energetically as to produce convulsions, the latter arrests the respiratory process and so rapidly accumulates carbonic acid in the blood, that its narcotic and sedative qualities soon overcome the excitement of the nervous and muscular structures, and the convulsive movement ceases. Again, some cause is brought to bear in such a way as to disturb or excite the elementary properties of the tissues, and thereby develope the phenomena of fever. The fever thus established is accompanied by a general diminution of the excretory actions. Hence, effete matter gradually accumulates in the blood until, by its depressing effects, it overcomes the morbid susceptibility, thereby reducing the febrile excitement to a point consistent with the return of secretion and healthy action. This may take from one to three weeks; but when accomplished, the fever is said to have been *self-limited*, or cured by the *efforts* of *nature.* We thus see clearly that *nature* in medical language, when not used merely as a cloak for ignorance, means simply the ordinary processes that take place in the living animal body; and the *curvative efforts* or *powers* of *nature*, the *vis medicatrix naturæ*, are the performance of one or more of these processes in such a manner as, either to remove the causes, or overcome the effects of disease. Having thus defined nature and her efforts, we will next inquire into the nature of *art* and her real relations to nature.

Dr. Holmes has defined Art, to consist in the use of medicine for the production of extraordinary abnormal impressions to relieve disease. And medicines, he defines to be *unnatural noxious* agents. These definitions, however, are only equalled in absurdity by his declaration that nature means a mental act of faith or *trust.* We should say that *disease* consisted in extraordinary and abnormal impressions and actions; and that *art*, as used in medical language, meant the use of such agents as would diminish or *remove*, such "extraordinary and abnormal impressions" and their results. In the illustrations already given we have seen that nature has three methods of resisting disease; First, by expelling the cause, as when offending matter is ejected by gastric or intestinal discharges or by increased secretions from the skin, kidneys, &c. Second, by increasing the action of one secretory organ sufficient to compensate for the diminished action of another. Third, by allowing the accumulation of such material in the blood, as by its narcotic or sedative qualities is capable of overcoming morbid excitement and favoring critical evacuations.

So the enlightened physician, the practitioner of true medical art, instead of using noxious agents to produce abnormal impressions, acts in strict imitation of nature's own processes, by endeavoring first of all to remove the noxious causes—second, to use one organ when necessary for the temporary relief of another which may be disabled by disease—and third, to bring into requisition such agents as will allay irritation or excitement when in excess, or prop up and sustain the properties and functions when too much depressed.

We thus see that the operations of nature herself; or in other words, the processes that take place in the human system when embarrassed by morbid impressions or disease, plainly indicate the use of agents to remove offending matter, either by evacuants, such as cathartics, diuretics, diaphoretics &c., or by antidotes to destroy its noxious qualities. And when the morbid actions do not cease with a removal of the causes from which they originated, nature points with equal clearness to the employment of sedatives, anodynes, or tonics, according to the nature of the morbid action.

The very existence of disease, is a confession on the part of nature, that she has been unable to resist the action of those noxious influences to which she has been exposed. And the true object of art, is to interpose timely and well directed assistance in her behalf, either by removing the evil influences, or lessening their effects on the organized structures, or by both. Hence the skill of the physician in the practice of his art, will be in direct proportion to the clearness with which he sees the exact nature and tendency of the morbid actions constituting disease, and the precision with which he adapts the application of remedial agents to the accomplishment of the special change indicated by the nature of the morbid action. If there is any truth in the foregoing observations, they show clearly that the physician is, what his name imports, namely, a student of nature in its most comprehensive sense. For he studies nature, not only, in her healthy manifestations, but also in her embarrassments, her deformities, and morbid conditions. And his *art*, instead of being antagonistic or opposed to nature, is in the truest sense of the word, her ally, her assistant, exerted only when she is embarassed, and in a direction to relieve such embarrassment.

Hence, the assumption of Dr. Holmes, and the school he represents, that nature and art stand in opposition to each other, is a fundamental error that vitiates all their reasoning. Equally faulty is the definition of medicines given by the same class of writers, and the distinction between food and medicine. The assertion that food embraces all substances capable of nourishing the tissues or supporting the functions of the organs; and that medicines are simply "unnatural or noxious agents," is neither explicit nor truthful. Will any respectable physiologist deny that caloric, electricity, oxygen, &c., are capable of supporting the various functions of the system, and yet will he pretend that these agents are food? Again, it is perfectly well known, that under certain circumstances, strychnine, the preparations of iron, of peruvian bark, &c., are capable of aiding very much in supporting the various functions of the system; but will any one pretend that these agents are food! Neither is it true that all medicines are "unnatural or noxious agents." Are not the gentian, the poppy, and the rheubarb, just as *natural* as the potatoe, the corn, or the wheat? And, when properly used, are they any more noxious? If improperly used, they may produce injurious or noxious effects, and so may a loaf of bread. The truth is that the human system is capable of being acted upon by three distinct classes of agents. The first embraces all such substances, whether solids, liquids, or gases, as are capable of being assimilated and converted into any part of the natural structures of the body. These constitute *food.*

The second, embraces those agents, which though not constituting a part of the living structures, are yet essential to the healthy performance of the various functional and organic actions, such as atmospheric oxygen, caloric, light, and electricity. These are the natural *stimuli* or *excitors* of action, and are essentially distinct from food.

The third class, embraces all such agents as are capable of modifying the properties and actions of the system, without being themselves assimilated. These are *medicines.*

Another fundamental error, that vitiates the reasoning of all that class of writers who delight to deify nature, and caricature art, consists in the assumption that all medicines or substances capable of affecting a well man injuriously, must also affect a sick man in the same direction. Thus, if a bleeding, a purge, or a blister, debilitates a person in health, the same effect must necessarily follow their administration to one affected with disease. To the observing and experienced practitioner, the fallacy of such a statement, is abundantly obvious; and yet it is well calculated to deceive the inexperienced and the non-professional. Those who assert such a doctrine, forget that the intense engorgement of the capillaries of an important organ like the lungs or brain, may so interfere with the performance of its function as to threaten a speedy and fatal exhaustion, while a timely bleeding sufficient to make a well man faint, would, by relieving the oppressed organ and restoring its function, positively add to the strength of the sick one. They seem to forget that all disease or morbid action, is accompanied by abnormal susceptibilities and diminished strength; and consequently that agents which might act positively injurious on organs with their normal susceptibility and action, might be exactly adapted to remove abnormal susceptibility and action, and thereby restore strength and health. Without pursuing these strictures further, we will briefly state a few rules which we think should govern the physician in the investigation and treatment of disease.

First, he should regard the living body, as a complex organization, composed of various delicately organized structures and organs, each designed to perform its own proper function, and all endowed with elementary properties that distinguish them from inorganic or dead matter.

Second, he should remember that the body so organized and endowed, is constantly surrounded by agents, ponderable and imponderable, so numerous and so changeable, that they are capable of influencing the properties and functions of its several structures, in every possible direction. Thus, some are capable of exalting the properties and inducing excited action; others of depressing the properties and inducing diminished action; while others still pervert the properties and functions, instead of simply exalting or depressing them.

Third, the physician should aim to make himself so familiar with the physiology and pathology of every structure and organ, that he is capable of analyzing the complex phenomena of disease, and of tracing accurately its location, its grade of action, its stage of progress, its tendencies, and its influence on other organs more or less remote from its own location.

Having done this, he should have such a knowledge of the Materia Medica, as will enable him to apply such remedies as are exactly adapted to the existing grade of morbid action. In doing this he will find use for depletives, sedatives, and anodynes to soothe pain and diminish excited action; for tonics and restoratives to prop up and sustain functions already impaired; and for alteratives and antidotes to correct perverted action, and neutralize noxious ingredients.

Following these rules and objects, the enlightened physician will neither become a skeptical advocate of expectancy; nor an indiscriminte administrator of heroic remedies; nor yet a dreamy advocate of some vague theory by which he attempts to reduce all disease to a unit, and all remedies to one principle of action, whether such theory be the *asthenia* of Brown and Todd, or the similia similiabus curantur of Hannemann. But he will become a true observer of nature, in health and disease; and will be ever ready to aid her when embarrassed by disease or morbid action, strictly in accordance with the dictates of an enlightened *common sense.*

